# Evidence that publication bias contaminated studies on visual event boundaries and anchoring effects

**DOI:** 10.1073/pnas.2320278121

**Published:** 2024-03-18

**Authors:** Gregory Francis

**Affiliations:** ^a^Department of Psychological Sciences, Purdue University, West Lafayette, IN 47907

Ongchoco et al. (1) presented evidence across seven pre-registered experiments that anchoring effects are attenuated when people cross a visual boundary. Repeatedly finding such evidence might seem to demonstrate a robust and reliable effect, but it actually undermines the conclusions of Ongchoco et al. because it is inconsistent with the modest power of their experiments.

Each experiment in Ongchoco et al. found an anchoring effect (valuation judgments are influenced by the magnitude of a previously observed number) when taking a walk through a virtual environment without a clear visual event boundary and a weaker (perhaps absent) anchoring effect when the virtual walk crossed a visual event boundary. The first analysis always produced a significant *t* test, while the second analysis always produced a significant interaction (*F* test). Due to random sampling, repeated tests should reject the null hypothesis with a frequency that reflects the underlying power of the experiments (2, 3). Table 1 shows the statistical properties of the experiments and the estimated probability (power) to find both significant effects for each study with the given sample size. The estimate supposes that each experiment reports means and SD that reflect the true population values and uses 100,000 simulated datasets to find the proportion of samples that satisfy both tests (4). The estimated power is low. For example, study 5 found barely significant outcomes for both the *t* test and the *F*-test. Given the size of the effects and the sample size, the probability that a random sample for this experiment would lead to significant outcomes for both tests is only 0.388.

**Table 1.** Statistical properties of the experiments in Ongchoco et al

	Sample size for each of four conditions	Statistics	Estimated power
Study 1	200	*t*(398) = 2.52, *P* = 0.012 *F*(1,796) = 4.49, *P* = 0.034	0.504
Study 2	200	*t*(398) = 2.32, *P* = 0.021 *F*(1,796) = 6.90, *P* = 0.009	0.577
Study 3	200	*t*(398) = 2.07, *P* = 0.039 *F*(1,796) = 7.01, *P* = 0.008	0.507
Study 4	200	*t*(398) = 2.44, *P* = 0.015 *F*(1,796) = 5.17, *P* = 0.023	0.530
Study 5	200	*t*(398) = 2.03, *P* = 0.043 *F*(1,796) = 3.98, *P* = 0.046	0.388
Study 6*	400	*t*(798) = 2.03, *P* = 0.043 *F*(1,1596) = 7.27, *P* = 0.007	0.487
Study 7	250	*t*(498) = 10.44, *P* < 0.001 *F*(1,996) = 7.01, *P* = 0.008	0.753
Joint power for all studies			0.011

Note: *The original manuscript gives the wrong degrees of freedom for study 6. The numbers here are correct.

As Table 1 shows, across the seven experiments in Ongchoco et al., most of the experiments have a power of around 0.5. The probability that seven independent experiments like these would all produce significant results is the product of the power estimates for each experiment: 0.011.

So how did Ongchoco et al. get such unusual data? Each experiment was pre-registered, which seems to rule out various types of p-hacking (5). However, pre-registration of experiments does not protect against publication bias. Perhaps Ongchoco et al. ran additional experiments that did not support their conclusions but did not report them in their article. Such publication bias misrepresents the scientific findings and renders their reported results uninterpretable. Thus, the conclusions reported in Ongchoco et al. should be considered as unsupported until the experimental results are independently replicated with much larger samples (4).
